# Feeding the Walls: How Does Nutrient Availability Regulate Cell Wall Composition?

**DOI:** 10.3390/ijms19092691

**Published:** 2018-09-10

**Authors:** Michael Ogden, Rainer Hoefgen, Ute Roessner, Staffan Persson, Ghazanfar Abbas Khan

**Affiliations:** 1 School of Biosciences, University of Melbourne, Victoria 3010, Australia; ogdenm@student.unimelb.edu.au (M.O.); u.roessner@unimelb.edu.au (U.R.); staffan.persson@unimelb.edu.au (S.P.); 2 Max Planck Institute of Molecular Plant Physiology, Potsdam-Golm 14476, Germany; hoefgen@mpimp-golm.mpg.de

**Keywords:** cell wall, nutrients, root system architecture

## Abstract

Nutrients are critical for plants to grow and develop, and nutrient depletion severely affects crop yield. In order to optimize nutrient acquisition, plants adapt their growth and root architecture. Changes in growth are determined by modifications in the cell walls surrounding every plant cell. The plant cell wall, which is largely composed of complex polysaccharides, is essential for plants to attain their shape and to protect cells against the environment. Within the cell wall, cellulose strands form microfibrils that act as a framework for other wall components, including hemicelluloses, pectins, proteins, and, in some cases, callose, lignin, and suberin. Cell wall composition varies, depending on cell and tissue type. It is governed by synthesis, deposition and remodeling of wall components, and determines the physical and structural properties of the cell wall. How nutrient status affects cell wall synthesis and organization, and thus plant growth and morphology, remains poorly understood. In this review, we aim to summarize and synthesize research on the adaptation of root cell walls in response to nutrient availability and the potential role of cell walls in nutrient sensing.

## 1. Introduction

Plants are the primary producers on earth, accounting for 80% of all living biomass, and provide us with food, feed, and shelter [1]. Nutrient availability controls how plants grow and thus their ability to produce biomass [2]. The levels of macro- and micro-nutrients in the soil, and the ability of plants to access them, therefore have major implications on agriculture and ecology [3]. To maximize acquisition of nutrients, plants adjust their growth and metabolic processes [4,5,6]. By deliberately limiting their chlorophyll content and rate of photosynthetic carbon fixation to coordinate their growth with resource availability, plants achieve optimal morphology to survive [3]. Cell division and cell elongation are the major factors that drive growth and morphology in plants. Unlike animal cells, plant cells are surrounded by a protective and supportive structure, referred to as the cell wall, which contributes the bulk of a plant’s biomass. Dynamic remodeling of this structure is crucial for plant cells to divide and elongate [7,8]. Because the cell wall dictates cell and tissue morphology, and as nutrient availability drives changes in plant growth, it follows that changes to the cell wall structure should, indirectly or directly, be controlled by the nutrients available to the plant. There is mounting evidence from gene expression data that cell walls are actively regulated in response to nutrient availability [9,10,11,12,13]. However, while it is clear that reprogramming of cell wall genes is essential for plant adaptations to nutrient status, knowledge relating to molecular mechanisms controlling these changes is just starting to emerge. In the agricultural context, plant biomass and nutrient availability are the two most important variables. Research in this field is critically important and has direct links to societal and economic benefits. In this review, we summarize the current understanding of how cell walls are dynamically regulated in response to nutrient availability and how cell wall defects cause changes to nutrient sensitivity. In order to understand the relationship between cell wall and nutrients, we first introduce the basic concepts of cell wall synthesis and structure, along with nutrient transport. Unless otherwise noted, examples presented are from the model plant *Arabidopsis thaliana* (Arabidopsis).

### 1.1. Cell Walls

Plant cell walls are highly dynamic structures. They are composed primarily of polysaccharides, including cellulose, pectins, hemicelluloses, and callose. In this review, we focus only on the areas of cell wall synthesis and regulation that are directly relevant to nutrient response. There are excellent reviews that cover key topics of cell wall synthesis and regulation [7,14,15,16,17,18,19].

#### 1.1.1. Cellulose

Cellulose is composed of β-(1→4)-d-glucan chains, which are crystallized into cellulose microfibrils through inter- and intra- molecular hydrogen bonds and Van der Waals forces. These microfibrils are the primary loadbearing polymers of cell walls and act as a framework for tethering and deposition of other wall components [19].

Cellulose is synthesized by CELLULOSE SYNTHASE A (CESA) catalytic subunits, which are organized at the plasma membrane in large multiprotein complexes called cellulose synthase complexes (CSCs). The CSC constitutes a heteromeric arrangement of 18 to 24 CESAs, with CESA1, CESA3, and a CESA6-like protein (CESA2, 5, 6 or 9) being required for primary cell wall synthesis [14,20]. Certain specialized cells, such as xylem tracheary elements, also synthesize a secondary cell wall that is deposited between the primary cell wall and the plasma membrane. During secondary cell wall synthesis, the CSC is comprised of CESA4, CESA7, and CESA8 [21,22]. Most secondary cell walls contain a significantly increased amount of lignins, which are hydrophobic aromatic polymers typically derived from phenylalanine [23].

Apart from their plasma membrane localization, CSCs are also localized at the Golgi apparatus, trans-Golgi network, small CESA compartments, or microtubule-associated cellulose synthase compartments [24]. These latter compartments may be involved in delivery or internalization of the CSCs [14]. During synthesis, newly formed cellulose microfibrils become entangled in cell walls through cross-linking with cell wall polymers. Further synthesis of cellulose pushes the CSCs forward along the plasma membrane [25,26]. CSC speed is therefore often used as a proxy for CESA catalytic activity. The direction of CSC movement, as well as its targeted delivery to the plasma membrane, is guided by cortical microtubules [27]. Several proteins are involved in guiding the CESAs along microtubules; their functions are necessary to maintain cellulose synthesis. These proteins include CELLULOSE SYNTHASE-MICROTUBULE UNCOUPLING (CMU), COMPANIONS OF CELLULOSE SYNTHASE (CCs), and CELLULOSE SYNTHASE INTERACTING PROTEIN 1(CSI1) [28,29,30]. Of particular note, CCs bind to CSCs and microtubules, and regulate cellulose synthesis under salt stress conditions by re-establishing the microtubule array following salt stress-mediated microtubule depolymerization [29]. KORRIGAN 1 (KOR1), an endo-1,4-β-d-glucanase, also functions in cellulose synthesis by interacting with the CSC at the plasma membrane and during intracellular trafficking [31]. Although the precise function of KOR1 is unknown, *kor1* mutants display reduced cellulose synthesis, and KOR1 is thought to play a role in relieving tensional stress generated during microfibril synthesis, or by releasing microfibrils from the CSC during cessation of cellulose synthesis [32]. COBRA and COBRA-like proteins encode glycosylphosphatidylinositol anchored proteins, and are involved in cellulose synthesis and modify cellulose crystallinity [33]. Finally, the chitinase-like protein homologs, CHITINASE-LIKE PROTEIN 1 (CTL1) and CTL2, bind cellulose and impair CSC activity at the plasma membrane [34,35,36]. Although the exact function of CTL1 and CTL2 is unclear, CTL1 colocalizes with CESAs during secretion to the apoplast [35,36].

#### 1.1.2. Pectins

Pectins are a diverse family of complex acidic polysaccharides that act as a hydrophilic gel in which other cell wall components are embedded. Pectin composition can vary widely in chain length and branching complexity; however, all pectins contain 1,4-linked α-d-galacturonic acid residues [19]. Pectins are synthesized in the Golgi, and require a minimum of 67 transferases, including glycosyltransferases, acetyltransferases, and methyltransferases, many of which remain unknown or uncharacterized. A large number of transferases may be required due to the many diverse linkages present in pectins [16]. Following synthesis, pectins are packaged into vesicles and trafficked to the plasma membrane for secretion to the cell wall [37]. In the cell wall, cellulose and pectins are closely linked via hydrogen bonds [38]. Pectin deposition and pectin modification play a central role in mediating cell growth [39]. Pectins are typically secreted to the cell wall in a highly methyl-esterified form, and cell wall-localized pectin methylesterases can act upon pectins to cleave methyl ester bonds to remove the methyl groups [40]. This results in the production of free carboxylic groups, which drastically alters the physical properties of pectins, where low levels of methyl-esterified pectins are typically associated with decreased cell wall extensibility and growth inhibition [41].

#### 1.1.3. Hemicelluloses

Similar to pectins, hemicelluloses are synthesized within the Golgi, packaged into vesicles, and secreted to the apoplast [42]. Hemicelluloses are characterized as molecules containing a backbone of β-(1→4)-linked xylose, glucose, or mannose. Mixed linked glucan (MLG) is a special hemicellulose containing a backbone of unbranched (1,3)- and (1,4)-linked b-glucosyl residues and is typically found in grasses, such as cereals [43], which contain distinct differences in cell wall composition compared to dicotyledons, like Arabidopsis [44]. Within the cell wall, hemicelluloses function by crosslinking cellulose microfibrils via hydrogen bonds [42]. Cosgrove (2014) [45] proposed a model, where xyloglucans, a hemicellulose, act as an adhesive layer between cellulose microfibrils to bundle them together at biomechanical hotspots, thus performing an important role in cell wall structure and integrity by maintaining a strong network of interconnected cellulose microfibrils.

#### 1.1.4. Callose

Although plant cells are surrounded by cell walls, they are also symplastically connected through plasmodesmata (PD) (analogous in function to gap junction in animal cells). These are symplastic channels that transverse the cell walls and connect the cytoplasm of adjacent cells [46]. For cell-to-cell transport of signaling molecules to occur through PD, cell wall composition is important, as the major regulation of PD transport is controlled by the deposition or removal of callose (β-1,3-glucan) in the cell walls surrounding PD. Callose deposition causes restriction of the symplastic channels, isolating the cells from each other and interrupting symplastic signaling [46]. Callose is also deposited in the cell plate in dividing cells, pollen tubes, roots, and can be induced by various biotic and abiotic stresses, including wounding [46,47,48].

#### 1.1.5. Suberin

Suberin is a hydrophobic lipid phenolic polyester comprised of aliphatic, glycerol, and phenolic monomers, with α-ω-dicarboxylic acids and ω-hydroxy acids being the main monomers [49]. Suberin precursors are transported from the endoplasmic reticulum to the plasma membrane for secretion to the apoplast, where suberin polymerization occurs; however, the pathways that mediate these processes remain largely unknown [49]. Suberin localizes to the cell walls of specialized cells, including seed coats and root endodermal cells. Suberin deposition can occur in response to various biotic and abiotic stresses. Most importantly, suberin is deposited to the inner surface of the primary cell wall of root endodermal cells to act as a barrier for diffusion of water and nutrients to the stele [49].

#### 1.1.6. Lignin

Lignin is a complex phenolic polymer typically deposited to secondary cell walls and commonly associated with woody tissues [50]. Lignin is mainly comprised of three monolignols (hydroxycinnamyl alcohols) synthesized from phenylalanine, including ρ-coumaryl, sinapyl, and coniferyl alcohols [23]. Within the cytoplasm, a complex array of reactions is required for monolignol biosynthesis, including deamination, hydroxylation, methylation, and reduction [50]. Monolignols are then transported to the plasma membrane and secreted to the cell wall, where polymerization and crosslinking occurs. Lignification induces unique physical characteristics to cells and tissues by enhancing rigidity through increased cross-linking of cell wall polysaccharides [51]. Lignin deposition is developmentally regulated within specialized cells and tissues, such as the endodermis and xylem [52]. Lignin deposition can also be induced in response to various biotic and abiotic stresses [53]. In the root endodermis, lignin is deposited in maturing cells, specifically to the radial and transverse domains of the cell wall, in a ring-shaped conformation known as the Casparian strip [54]. This acts as a barrier to the apoplastic diffusion of water and nutrients and is important in maintaining nutrient homeostasis. Characteristic hydrophobic properties of lignin are also essential for efficiently transporting water and minerals in water-conducting xylem cells [55].

#### 1.1.7. Structural Proteins and Enzymes

A wide variety of structural proteins and enzymes is required to remodel structures or interactions of cell wall components, leading to wall extensibility and loosening. These proteins include xyloglucan hydrolases, β-1,4-glucanases, peroxidases, extensins, and expansins [56]. Their expression is regulated by a variety of abiotic stresses, including drought and salt stress. Several studies found that these proteins regulate cell wall stiffness through the cross-linking of different polymers. Consequently, ectopic expression of several cell wall modifying proteins leads to abiotic stress tolerance or hyper-susceptibility [57,58].

## 2. Importance of Cell Walls in Nutrient Transport

In addition to carbon dioxide and water, both of which are required for photosynthetic production of carbohydrates, plant growth depends on nutrient acquisition from the soil. Balanced proportions of a range of macronutrients (nitrogen (N), phosphorus (P), potassium (K), and sulfur (S)), and micronutrients (iron (Fe), copper, molybdenum, selenium, zinc, and others) are essential for optimal growth and crop yield. Plant growth under nutrient-deficient conditions typically results in nutrient-specific phenotypes, including an overall reorganization of root system architecture (RSA), and oftentimes, growth inhibition [59].

**Figure 1:**
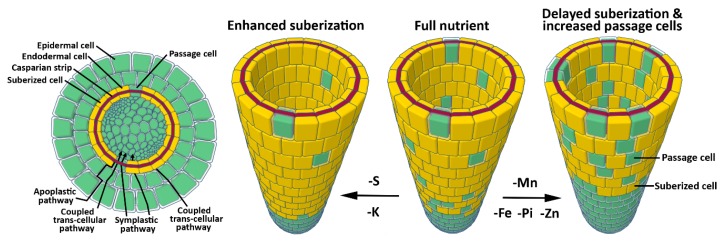
Plasticity in the permeability of endodermal cell walls in response to nutrient imbalance. (**Left** Panel) Cellular schematic cross section of a fully differentiated Arabidopsis root. Suberized endodermal cells (yellow) and the lignified endodermal Casparian strip (red) block nutrient transport via the apoplastic and coupled-trans-cellular pathways. Passage cells are unsuberized endodermal cells that allow symplastic and coupled trans-cellular transport. (**Right** Panel) Magnified view of the endodermal cell layer under varying nutrient availability. K and S deficiency causes an increase in suberization, while deficiency in Fe, Zn, and P causes a delay in suberization and increased number of passage cells. Mn deficiency also delays suberization, but its impact on passage cell occurrence is unknown. This plasticity in root cell wall permeability is an excellent demonstration of how plants modulate nutrient transport in response to varying nutrient concentrations in soil. Fe: iron; K: potassium; Mn: manganese; Pi: phosphate; S: sulfur; Zn: zinc. Schematics were modeled using the online software Tinkercad (www.tinkercad.com).

Nutrients and water are absorbed at the root-soil interface and are translocated from the soil to the stele via three distinct pathways: An apoplastic pathway, a symplastic pathway, and a coupled transcellular pathway [60]. Water and nutrients freely diffuse through the apoplastic pathway in an unregulated manner. The symplastic pathway is characterized by nutrient movement between cells via PD, with the coupled-transcellular pathway describing nutrient movement in and out of the cells, mediated by activity of influx and efflux carriers (Figure 1) [60]. Cell walls play a key role in regulating nutrient transport between soil and the stele. Casparian strips are highly lignified, hydrophobic cell walls that encompass the radial and transverse domains of root endodermal cells in the zone of maturation. Casparian strips fill the cell wall between each endodermal cell, creating an apoplastic barrier to water and nutrient diffusion. Within the maturation zone, nutrients must therefore move through the symplastic and coupled transcellular pathways to bypass Casparian strips [61]. Following Casparian strip formation, suberin is deposited to the inner surface of the primary cell wall, entirely enveloping each endodermal cell [49,60]. The so-called suberin lamellae initiates along patches of endodermal cells apical to the Casparian strip initiation site, eventually displaying homogenous cell wall distribution across the endodermis at increasing distances from the root tip. This barrier is crucial in limiting the diffusion of water and nutrients across the endodermis, thus protecting the plant from nutrient influx at toxic levels, or nutrient leaching from the stele [62]. Suberin production is tightly regulated by ethylene and abscisic acid (ABA) signaling, and its synthesis and deposition is highly plastic in response to nutrient availability. Moderate deficiency in Mn, Fe, and Zn leads to a delay in suberization, while K- and S-deficiency results in increased suberization [62]. This suggests a nutrient management strategy by the plant that modulates bidirectional movement of nutrients across the endodermis. In agreement with this, targeted degradation of suberin leads to suppression of the mutant phenotype in the Fe uptake mutant *irt.* Moreover, suberin degradation in the endodermis enhances the S deficiency phenotype of S uptake mutants for *SULFATE TRANSPORTER 1* (*sultr1;1sutr1;2*) and leads to K deficiency in plants [62], demonstrating the high plasticity of suberin regulation as an adaptive response to various nutritional cues. The suberization response to nutrient availability is a new field of research that will benefit our understanding of the regulation of nutrient homeostasis within plants.

Within the root zone of continuous suberization, randomly distributed cells, known as passage cells, lack suberin deposition (Figure 1) [61,63,64]. These cells are closely associated with the xylem pole. They lack secondary cell wall deposition and their development is mediated by repression of cytokinin signaling in the root apical meristem (RAM) (Figure 1) [64]. The exact role of passage cells is unclear; however, it is thought that they facilitate water and nutrient transport or cell communication in older parts of the root [61,63,64]. In line with this, expression of the phosphate (Pi) efflux protein family member *PHOSPHATE1* (*PHO1*), which is typically present in pericycle cells, expands into the endodermal passage cells [64]. Furthermore, Pi, Fe, and Zn deficiency simultaneously reduces suberization and increases passage cell occurrence [64]. Dynamic regulation of passage cell occurrence in response to nutritional status and expression of nutrient transporters in these cells supports the hypothesis that passage cells are involved in nutrient transport. Overall, these results suggest that plants tightly regulate nutrient transport across the root by modulating nutrient flux carriers and cell wall permeability (Figure 1).

## 3. Influence of Nutrient Availability on Cell Wall Composition

### 3.1. Nitrogen

All plants utilize N in the form of ammonium (NH_4_^+^) and nitrate (NO_3_^−^). Various groups have investigated the transcriptomic response of Arabidopsis plants to N availability. These studies have mainly relied upon NO_3_^−^ induction or deprivation, followed by RNA extraction and Affymetrix microarray analyses [9]. Few studies specifically highlight the expression of cell wall related genes; however, it was noted that “glucose catabolic process” is one of the most consistent biological functions associated with NO_3_^−^ response. Genes involved in trichoblast differentiation, which contain many cell wall related genes, were particularly enriched in NO_3_^−^ responsive genes [9]. Additionally, a co-expression analysis of NO_3_^−^ transporters, using the ATTED-II database, revealed that they are co-expressed with many cell wall related genes [65]. However, caution should be exercised in interpreting this data, as a recent meta-analysis of NO_3_^−^-response transcriptomic data revealed a striking inconsistency in the reproducibility between different studies, noting that the common number of differentially expressed genes between any two experiments is only 6.7% [9]. This variation in expression patterns is likely due to redundancy of large gene families, developmental differences, and changes in experimental setups between labs, as gene expression is highly sensitive to environmental conditions and plant age. This is highlighted in plants that were not subjected to changes in NO_3_^−^ availability, but showed gene induction resulting from other stress related to simply moving the plants from one solution to another [66]. Transcriptomic assays would greatly benefit from a standardized experimental setup, with detail paid to the nutrient composition of media, lighting levels, plant handling and transfer between media, and the time at which tissue is harvested following treatment. By adopting a standardized experimental setup, future transcriptomic results may lead to increased reproducibility in gene expression trends.

Under N-limiting conditions, plants adapt their RSA to maximize N acquisition by exploiting a larger soil surface area. This is mostly achieved by the elongation of lateral roots (LRs), whereas high N concentrations result in an inhibition of LR growth, with little effect on primary root (PR) growth [59,67]. In crop plants, N status affects stem mechanical strength and disease resistance. These traits are regulated by cell wall organization and strength, suggesting that cell walls are modulated in response to N status. Expression of genes involved in biosynthesis of both lignin and cellulose was significantly reduced in response to high N in rice [68,69]. In support of this finding, cell wall analysis of roots showed that high N leads to a significant reduction of cellulose and lignin. This reduction in cell wall components is accompanied by reduced stem mechanical strength, increased lodging, and reduced disease resistance [68,69,70]. Similar to roots, expression of cellulose and lignin synthesis genes is reduced in filling seeds in response to high N. This leads to reduced accumulation of both cellulose and lignin in seed endosperms [71]. In line with this observation, N deficiency leads to an increase in cellulose content in the roots of rice plants [72].

In Arabidopsis, intact cell walls are essential for the adaptation of RSA in response to N availability, as genetic mutants with altered cell wall organization show impaired RSA response to N availability. Cellulose-deficient mutant roots are hypersensitive to high (60 mM) KNO_3_ [73]. When grown under high N conditions, *cesa3*, *cesa6*, and *ctl1* mutants show a drastic reduction in PR length, an increase in root hair and LR density, and swollen roots. Further investigation of *ctl1* revealed the same hypersensitive root response to high NaCl, KCl and CaCl_2_, whereas no response was observed under high PO_4_, SO_4_, mannitol, or sorbitol, suggesting a hypersensitive response to chloride ions [35]. Hypersensitivity to chloride in conjunction with NO_3_^−^ is not unexpected, as multiple NO_3_^−^ transporters are also known to function in chloride transport [74]. Furthermore, under high KNO_3_, *ctl1* and *cesa3* mutants displayed ectopic lignin deposition in the endodermis, whereas *cesa6* and *kor1* exhibited only minor ectopic lignin deposition compared with wild-type controls [73]. If cellulose reduction occurs in Arabidopsis under high N availability (as also observed in rice), this could explain the hypersensitive root phenotype of cellulose deficient mutants when subjected to high N, resulting from an additive effect due to high N-mediated reduction in cellulose in an already cellulose deficient mutant. These results demonstrate the complex response to N availability, inferring a dynamic regulation of cell wall synthesis and remodeling. However, the molecular mechanisms by which N status affects cell wall biosynthesis and organization are unknown.

### 3.2. Phosphorus

In a recent study of Pi-responsive genes, a detailed transcriptomic analysis using wild-type and mutants for *LOW PHOSPHATE RESPONSE 1* (*LPR1*) and *LPR2*, and *PHOSPHATE DEFICIENCY RESPONSE 2*, which are required for Pi response, highlighted a robust Pi-dependent regulation of cell wall related genes [75]. These genes were sorted into four main groups, which included pectin modification, cell wall relaxation, hemicellulose/cellulose modification, and carbohydrate hydrolytic enzymes; however, it is unknown how these changes in expression impact growth. Additional studies in wild-type plants noted similar changes in expression of cell wall related genes in response to Pi starvation [10,13,76,77]. A transcriptomic analysis for seedlings subjected to Pi-deficiency revealed differential regulation of the largely uncharacterized *CELLULOSE SYNTHASE-LIKE B5* (*CSLB5*) [10]. Further investigation revealed that *cslb5* mutants produce shorter root hairs compared to wild-type under Pi-deficiency, suggesting a role of CSLB5 in Pi response [10]. The exact function of the CSLB family is unknown; however, it is thought that members are involved in the synthesis of β-1,4-linked cell wall polysaccharides [78]. Interestingly, *CSLB5* is expressed in roots, which goes in line with its likely function in regulation of RSA in response to Pi starvation [78].

**Figure 2:**
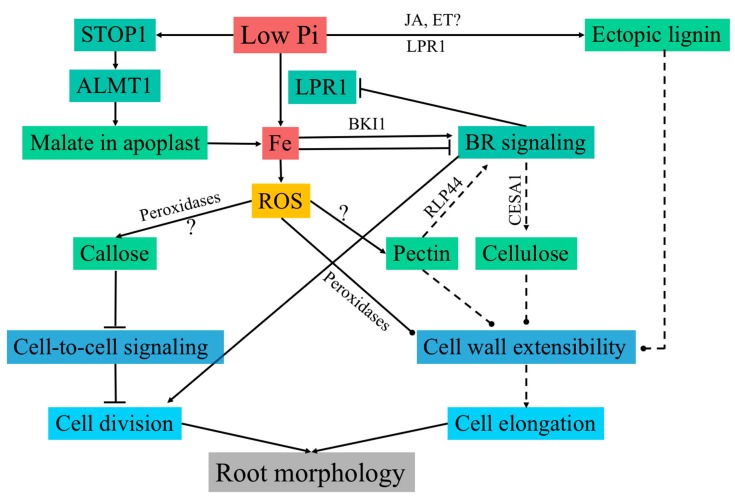
Model of cell wall regulation in response to Pi starvation. Low Pi causes LPR1-dependent Fe accumulation in the apoplast, which is required for the inhibition of primary root growth. Low Pi also leads to activation of the transcription factor STOP1 through an unknown mechanism. STOP1 induces the malate transporter ALMT1 by directly binding to its promoter. Subsequently, ALMT1 secretes malate in the apoplast, which is required for Fe accumulation in the transition zone. Apoplast-localized LPR1 expresses ferroxidase activity and is thought to generate ROS via Fe redox cycling. Cell-type-specific ROS generation is required for callose deposition and impairs symplastic connectivity, leading to inhibition of cell division in the RAM. In a feedback regulation, Fe modulates LPR1 expression via BR signaling in a concentration-dependent manner. Low Fe concentration activates, and high Fe concentration inhibits, BR signaling by translational regulation of BKI1, which is a key inhibitor of BR signaling. BR signaling is known to regulate cellulose synthesis by the transcriptional and post-translational regulation of CESAs. However, it is unknown whether cellulose synthesis is affected in response to low Pi. Additional cell wall modifications include ectopic lignin deposition, possibly via changes in jasmonate and ethylene signaling, peroxidase-dependent cell wall stiffening, and cell-type-specific pectin deposition. Changes in pectin are known to trigger BR signaling via RLP44 to maintain cell wall integrity. Dual control of BR signaling through pectin and Fe may be required to achieve essential changes in cell walls required for growth modulation in response to low Pi, while maintaining cell wall integrity. Cell wall modifications through changes in cellulose, pectin, and lignin, impact cell wall extensibility. These modifications could be responsible for the rapid inhibition of cell expansion in response to low Pi. Solid lines denote known interactions and dashed lines denote inferred interactions. Arrows are positive interactions and terminated lines indicate negative interactions. Round heads suggest interactions responsible for a change in cell wall architecture. STOP1: SENSITIVE TO PROTON TOXICITY 1; ALMT1: ALUMINUM-ACTIVATED MALATE TRANSPORTER 1; ROS: reactive oxygen species; LPR1: LOW PHOSPHATE RESPONSE 1; BR: brassinosteroid.

Under low Pi conditions in Arabidopsis, PR growth is arrested, while growth of secondary roots and root hairs is promoted [59,79]. PR growth arrest is mainly a result of rapid reduction in root cell elongation followed by an arrest of cell division in the stem cell niche. These changes in the RSA are dependent on the external supply of Fe. This suggests an indirect surveillance of Pi status via its interaction with transition metals [80]. During Pi deficiency, LPR1, which is a cell wall localized ferroxidase, modulates Fe deposition in the cell walls of elongating cells and the stem cell niche [48]. Interestingly, in these cells, Fe deposition is promoted by malate secretion to the cell walls. Under low Pi conditions, the transcription factor SENSITIVE TO PROTON TOXICITY 1 (STOP1) targets the malate efflux channel *ALUMINUM-ACTIVATED MALATE TRANSPORTER 1* (*ALMT1*), triggering malate secretion to the cell walls of the root transition zone. Malate deposition promotes Fe^3+^ accumulation via LPR1 activity in the elongating cells [81,82], coinciding with the generation of reactive oxygen species (ROS) and callose deposition [48]. Cell type-specific callose deposition disrupts symplastic communication through plasmodesmata as shown by impaired movement of the transcription factor SHORT ROOT between the stele and the endodermis [48]. Symplastic communication is essential for meristem maintenance, and its disruption is likely the main factor leading to root meristem exhaustion in response to low Pi (Figure 2) [83]; however, the mechanism behind the rapid inhibition of cell elongation, which occurs well before meristem size reduction, is unclear [48,81]. LPR1 mediated Fe accumulation leads to the generation of ROS, which coincides with the initial sites of Fe deposition in the apoplast [48]. Ferroxidase activity of LPR1 leads to Fe^3+^ redox cycling in the cell wall, which creates an overall oxidative environment and ROS generation [48]. ROS may serve as a substrate for the cell wall localized class III peroxidases to catalyze cross-linking between cell wall components (Figure 2) [18], leading to cell wall stiffness and reduced extensibility. Atomic force microscopy revealed an increase in cell wall stiffness in the elongation zone within 30 min following seedling transfer to Pi-deficient media, with the change in stiffness being dependent on the activity of class III peroxidases [81]. Pharmacological inhibition of peroxidases restored root growth in response to low Pi, confirming that increased cell wall rigidity is linked to inhibited root growth under Pi-deficient conditions [81]. Changes in cell wall stiffness need to happen in conjunction with changes in cell turgor in order to coordinate cell expansion [84,85]. This may be achieved through conformational changes in lipids [86], which typically result in changes in cellular trafficking and vacuolar organization [87,88], which are crucial factors for the regulation of intracellular turgor [85].

Varying Fe concentration leads to activation or inhibition of the brassinosteroid (BR) signaling pathway, which in turn regulates the expression of *LPR1* [89]. This feedback loop may be important in mediating distinct root morphologies as an adaptive response to soil microenvironments with varying concentrations of either Pi or Fe [89]. *BRASSINAZOLE RESISTANT 1* (*BZR1*), a key regulator of BR mediated regulation of gene expression [90], represses the expression of LPR1 by directly binding to its promoter [89]. In line with this, BZR1 constitutive mutants (BZR1-D) phenocopy the *lpr1* root phenotype in response to low Pi [91]. Low Fe activates, and high Fe inhibits, BR signaling via translational modulation of its key repressor brassinosteroid kinase inhibitor 1 (BKI1) through an unknown mechanism [89]. Fe concentration-dependent modulation of BR signaling is required for the root growth response in low Pi conditions (Figure 2). Interestingly, BR signaling is known to play a role both in cell proliferation and cell elongation via changes in cell wall composition. Mutants affected in the BR signaling pathway show significant transcriptional changes to various cell wall remodeling enzymes and structural proteins [92]. More importantly, BRASSINOSTEROID INSENSITIVE 2 (BIN2), a key inhibitor of the BR pathway, directly phosphorylates CESA1, leading to a decrease in CESA motility and crystalline cellulose synthesis [93]. BES1 and BZR1, which are the key transcription factors in the BR pathway, positively impact cellulose synthesis by directly binding to *CESA* promoters [94]. Along with the direct regulation of cellulose synthesis, BR is known to influence microtubule rearrangement [95], which is required to guide CSCs during cellulose synthesis. CSI1, CMU, and CCs are the proteins required for the association of CSC with microtubules [28,29,30]. Investigation of these proteins in context of BR and phosphate starvation response may prove to be an important tool in investigating changes in cellulose synthesis in low Pi stress. Overall, the BR pathway positively impacts cellulose synthesis, and it could be a strong candidate for the regulation of cellulose in response to low Pi.

The cellulose content of rice changes in responses to low Pi [96]; however, it is unknown whether Pi deficiency affects cellulose content in Arabidopsis. Furthermore, rice mutants affected in *CELLULOSE SYNTHASE-LIKE F6* (*CSLF6*) show a constitutively active Pi starvation response and increased Pi transport, even when seedlings are grown on high Pi [97]. CSLF6 is involved in the synthesis of MLG, which typically affects cell wall strength and cell expansion [43]. Interestingly, MLG binds to cellulose in vitro, forming a thick hydrogel at the surface of adsorption [98]. It is suggested that MLG may act as a transient binding surface where other cell wall polysaccharides can attach during periods of cell expansion [43]. Activation of the Pi starvation response in *cslf6* mutants implies an interdependent regulation of cell wall composition and Pi signaling.

Pi deficiency also leads to lignin accumulation in root cell walls; however, lignin deposition was not observed in the *lpr1lpr2* double mutant, suggesting that lignin deposition may be regulated by the same pathway as callose deposition under Pi-deficiency [99]. Lignin deposition is typically induced due to defects in cell wall integrity through the action of jasmonates and ethylene [100,101]. Both jasmonates and ethylene are induced in response to low Pi and possibly mediate the observed ectopic lignin deposition [102,103]. Lignin associates with cellulose and is involved in cell wall strengthening, implicating it in reduced extensibility of cell wall in low Pi conditions [104]. Under Pi-deficiency, the concentration of unesterified pectins significantly increases in cell walls in the elongation zone and the RAM, with a notable increase at the quiescent center and the border between the meristem and the stem cell niche [75]. The sites of increased pectin deposition colocalize to the sites of Fe^3+^ accumulation and callose deposition, indicating that pectins may play a role in cell wall organization in the root elongation inhibition response. Free carboxyl groups in unesterified pectins have a high affinity to bind to Fe^3+^, Al^3+^, and Ca^2+^, and pectins are known to dimerize via Ca^2+^-pectate crosslinked complexes called egg-boxes [105]. Increase in egg-boxes causes cell wall stiffening and reduced growth [106]. It is tempting to speculate that the so-called egg-box structures can be formed with the Fe^3+^, leading to an increase in cell wall stiffness. In addition, Fe binding with pectin may promote the release of Pi from Fe-Pi complexes during low Pi stress [107], which would be a fascinating adaptation to maximize Pi absorption. Interestingly, changes in pectin esterification are also known to trigger BR signaling via RECEPTOR-LIKE PROTEIN 44 (RLP44), which is required for the feedback regulation of cell wall modifications and coordination developmental output with cell wall integrity [108]. Dual control of BR signaling via pectin composition and Fe is perhaps required to modulate cell wall composition, driving RSA organization, while maintaining cell wall integrity.

Data summarized here demonstrate that cell wall modulation is essential for root response to low Pi, and that significant progress has been made in dissecting the complex genetic networks involved in this process; however, challenges remain in linking Fe accumulation and ROS generation with the modulation of cell wall chemistry. For example, what molecular mechanisms and pathways are responsible for recruiting callose synthesizing enzymes to the sites of Fe accumulation?

### 3.3. Other Nutrients

Apart from N and P, little is known about cell wall regulation by other nutrients. A study by Armengaud et al. (2004) [11] revealed that cell wall related genes, including extensins, xyloglucan glucosyltransferases, arabinogalactan proteins, and peroxidases, were enriched in genes that respond to low potassium (K). A meta-analysis of transcriptomic studies revealed that cell wall related genes are enriched in low S responsive genes [12]. As observed in the meta-analysis for genes responding to NO_3_^−^ availability, the reproducibility in consistent gene response was low between the datasets, with only 418 (20%) of the differentially expressed genes shared by a minimum of two experiments. It was found that the gene ontology term “cell wall” had a two-fold enrichment, genes coding apoplast-localized proteins were over-represented, and a predicted glycosyl hydrolase (At3g60140) was significantly up-regulated across all microarray datasets, implicating an S-dependent regulation of cell wall related genes.

The micronutrient boron forms so-called borate bridges by crosslinking the pectin polysaccharide rhamnogalacturonan-II [109]. These crosslinks modify cell wall porosity, mechanical properties, and are critical for plant growth. Consequently, boron deficiency leads to highly de-structured cell walls and severe growth defects [109].

As we understand more of the complex genetic networks in cell wall synthesis, it will be possible to identify more genes that regulate these networks in response to nutrient availability. A combination of in vivo and in vitro methods and systems biology approach [110] revealed a complex transcriptional network regulating secondary cell wall synthesis. Interestingly, this network contains several genes that are transcriptionally regulated in response to N, Fe, and S imbalance, suggesting that secondary cell wall synthesis is regulated by nutrient availability. Investigations revealed that the transcription factor REVOLUTA (REV) has a number of upstream factors that are regulated by Fe deprivation, suggesting that REV plays an important role in regulating secondary cell walls during Fe deficiency. Further experiments demonstrated that REV is a key transcription factor involved in the regulation of lignin biosynthesis in response to Fe deficiency [110]. These results demonstrate that cell wall synthesis is tightly regulated in response to nutritional cues and is underpinned by complex genetic networks.

## 4. Conclusions and Perspectives

Cell wall synthesis, composition, and remodeling is crucial for the meticulous modulation of RSA in response to nutrient imbalance. For example, Fe-mediated callose deposition in the cell walls of the root meristem and elongation zone is crucial for adapting the RSA in response to P deficiency (although it remains unclear how Fe regulates callose deposition in roots) [46]. Moreover, how other cell wall polymers are modulated in response to P imbalance is unknown. Modulation and reorganization of other cell wall polymers is expected, as a drastic reduction in cell division and cell elongation is observed shortly after exposure to low Pi. Apart from P, imbalances in other nutrients results in RSA reorganization [57], which is likely mediated by regulation of cell wall synthesis and/or architecture. This hypothesis is mainly supported by transcriptomics data due to technical advances, simplicity, and reduced cost of this type of analysis. It will be crucial for future studies to investigate changes in cell wall chemistry and cell wall composition using polymer-specific antibodies and mass spectrometry to understand the role of cell walls in nutrient-specific RSA modulation.

Experiments with rice show that cell wall polymers are reduced in response to excess N, which is mediated through transcriptional regulation of genes involved in cell wall biosynthesis [68,69]. It can be expected that cell walls are regulated in a similar manner in Arabidopsis given the sensitivity of cellulose deficient mutants to high N and Cl. If this is the case, Arabidopsis could prove to be a powerful tool for dissecting the molecular mechanisms of N- and Cl-mediated cell wall regulation. Investigating the relationship between N availability and cell wall regulation is important in an agricultural context, as high N directly affects important agronomical traits, such as lodging and disease resistance, through cell wall changes.

Researchers typically apply a reductionist approach by modulating the concentration of a single nutrient. However, in the natural environment, plants are typically exposed to changes in various nutrients simultaneously. Emerging evidence highlights the complexity and diversity of growth responses by subjecting plants to multiple nutrient deficiencies simultaneously, where the output is not an additive effect of each individual nutrient deficiency phenotype, but rather a unique phenotype altogether [111]. As soils contain a heterogeneous distribution of nutrients, and in order to understand their complex response pathways, it will be important to study multiple concurrent changes in nutrient concentrations.

Taken as a whole, the recent works referenced here demonstrate that cell wall synthesis, composition, and remodeling are crucial for both the transport and signaling of important nutrients. A robust body of knowledge now exists about how plants control water and nutrient transport across the root by manipulating cell wall permeability. It is clear that Arabidopsis root cell walls are highly plastic in response to the nutritional status of the soil [58]. It can be expected that this plasticity in root permeability helps plants to locally adjust nutrient transport in soil microenvironments with varying nutrient concentrations. Knowledge gained in this field has significant potential for engineering crops with increased nutrient use efficiency by tailoring RSA and endodermis permeability in accordance with soil nutritional status, while also improving growth and disease resistance.
